# Subunits of ARID1 serve as novel biomarkers for the sensitivity to immune checkpoint inhibitors and prognosis of advanced non-small cell lung cancer

**DOI:** 10.1186/s10020-020-00208-9

**Published:** 2020-08-13

**Authors:** Dantong Sun, Lu Tian, Yan Zhu, Yang Wo, Qiaoling Liu, Shihai Liu, Hong Li, Helei Hou

**Affiliations:** 1 Precision Medicine Center of Oncology, the Affiliated Hospital of Qingdao University, 59 Haier Road, Qingdao, 266000 Shandong China; 2 College of Environmental Science and Engineering, Ocean University of China, Qingdao, 266100 China; 3 Department of Medical Oncology, the Municipal Hospital of Qingdao, Qingdao, 266000 China; 4 Department of Thoracic Surgery, the Affiliated Hospital of Qingdao University, Qingdao, 266000 Shandong China; 5 Department of Medical Oncology, Qingdao West Coast New Area Central Hospital, Qingdao, 266555 China; 6 Medical Animal Laboratory, the Affiliated Hospital of Qingdao University, Qingdao, 266000 Shandong China; 7 State Key Laboratory of Oncogenes and Related Genes, Shanghai Cancer Institute, Renji Hospital, Shanghai Jiaotong University School of Medicine, Shanghai, 200032 China

**Keywords:** ARID1A, ARID1B, NSCLC, Immune checkpoint inhibitors, Prognosis

## Abstract

**Introduction:**

Patients with advanced non-small cell lung cancer (NSCLC) benefit from treatment with immune checkpoint inhibitors (ICIs). Biomarkers such as programmed death-ligand 1 (PD-L1), the tumor mutational burden (TMB) and the mismatch repair (MMR) status are used to predict the prognosis of ICIs therapy. Nevertheless, novel biomarkers need to be further investigated, and a systematic prognostic model is needed for the evaluation of the survival risks of ICIs treatment.

**Methods:**

A cohort of 240 patients who received ICIs from the cBioPortal for Cancer Genomics was evaluated in this research. Clinical information and targeted sequencing data were acquired for analyses. The Kaplan-Meier plot method was used to perform survival analyses, and selected variables were then confirmed by a novel nomogram constructed by the “rms” package of R software.

**Results:**

Seven percent of the NSCLC patients harbored *ARID1A* mutations, while 4% of the NSCLC patients harbored *ARID1B* mutations. Mutations in *ARID1A* and *ARID1B* were confirmed to be associated with sensitivity to ICIs. Patients harboring these mutations were found to have a better response to treatment (*ARID1A*: *P* = 0.045; *ARID1B*: *P* = 0.034) and prolonged progression-free survival (*ARID1B*: *P* = 0.032). Here, a novel nomogram was constructed to predict the prognosis of ICIs treatment. Elevation of the TMB, enhanced expression of PD-L1 and activation of the antigen presentation process and cellular immunity were found to be correlated with *ARID1A* and *ARID1B* mutations.

**Conclusion:**

ARID1A and ARID1B could serve as novel biomarkers for the prognosis and sensitivity to ICIs of advanced NSCLC.

## Introduction

Immune checkpoint inhibitors (ICIs) for cancer treatment have proven to be a great breakthrough in the past decade. In non-small cell lung cancer (NSCLC), ICIs have achieved convincing efficacy and tolerable safety in single-agent therapies or combined treatments and significantly prolonged the overall survival (OS) of patients (Leighl et al. 2019; Reck et al. 2019; Mok et al. 2019; Ready et al. 2019; Horn et al. 2017). During trials evaluating ICIs, several biomarkers have been found to be related to sensitivity to ICIs treatment, including high programmed death-ligand 1 (PD-L1) expression (Mok et al. 2019; Ready et al. 2019; Ott et al. 2019), a high tumor mutational burden (TMB) (Ready et al. 2019; Ott et al. 2019) and mismatch repair (MMR) deficiency (Mandal et al. 2019). In contrast, NSCLC patients harboring mutations of *epidermal growth factor receptor* (*EGFR*) and/or *anaplastic lymphoma kinase* (*ALK*) might not benefit from ICIs treatment (Gainor et al. 2016; Haratani et al. 2017). However, the reliability of these biomarkers in predicting the prognosis of ICIs treatment in NSCLC, especially the progression-free survival (PFS) of NSCLC patients, remains unclear, and at the same time, multivariate analyses of the sensitivity to ICIs involving the biomarkers described above and the genomic signatures confirmation of patients are necessary because of the heterogeneity and high somatic mutation rate of NSCLC cells. A hazard model for the prognosis of ICIs treatment is urgently needed.

The roles of switch/sucrose nonfermenting (SWI/SNF) chromatin remodeling complexes in a variety of biological processes during cell growth and development, including DNA replication, gene expression and cell differentiation, are essential (Wang et al. 2004; Zhang et al. 2014). These complexes are dysregulated in various cancer types (Huang et al. 2015). Canonical BRG1/BRM-associated factor (BAF), which is one of the three assembled SWI/SNF chromatin remodeling complexes (Naito et al. 2019), mainly consists of AT-rich interactive domain 1A/1B (ARID1A/1B) and DPF2 subunits (Michel et al. 2018; Mashtalir et al. 2018). According to the latest studies, ARID1A deficiency may be a novel biomarker for cancer immunotherapy (Shen et al. 2018; Jiang et al. 2020), and patients with ARID1A deficiency could benefit from ICIs treatment (Okamura et al. 2020). Nevertheless, the role of ARID1B, which serves as the other subunit of ARID1 together with ARID1A, in the sensitivity to ICIs treatment is unknown. Therefore, we performed this study of a previously published cohort of patients (Rizvi et al. 2018) to clarify the role of the ARID1 subunits in the prognosis of ICIs treatment among advanced NSCLC patients and the relationships between the ARID1 subunits with the tumor immune microenvironment (TIME) or other factors related to ICIs sensitivity and to establish a prognostic model for ICIs treatment of advanced NSCLC.

## Methods

### Study patients

Using the data derived from the cBioPortal for Cancer Genomics, 240 advanced NSCLC patients who received ICIs treatment and were described in previously published work (Rizvi et al. 2018) were involved in this research. The involved patients were treated with either anti-PD-(L)1 therapy (pembrolizumab) alone or in combination with anti-cytotoxic T-cell lymphocyte-4 (anti-CTLA-4) therapy (pembrolizumab+ipilimumab). Basic clinical information was collected from this cohort of patients, including age, sex and smoking history. The investigators used Response Evaluation Criteria in Solid Tumors (RECIST) version 1.1 to assess the efficacy of ICIs treatment, and PFS was assessed as the time that patients remained responsive to treatment (exhibited a complete response [CR], a partial response [PR] or stable disease [SD]). All involved patients gave their consent for the examination of targeted sequencing data, and tumor tissue samples from 86 patients were sent for PD-L1 expression assessment.

### Survival analyses and nomogram construction

The Kaplan-Meier plot (KM plot) method was used for univariate analyses of the involved patients, and the log-rank tests were used to detect significant differences. In addition, an online tool was used to perform survival analyses for all NSCLC patients (Gyorffy et al. 2013). Variables selected by the univariate analyses were used for the construction of a nomogram. The “rms” package of R software version 3.1.2 (The R Foundation for Statistical Computing, Vienna, Austria) was used to construct the nomogram. Harrell’s C-indexes ranging from 0.5 (no discrimination) to 1 (perfect discrimination) were used for the verification of discrimination (Bandos et al. 2009), and a visual calibration plot was used for the verification of calibration (Jin et al. 2017). Bootstrap analyses with 1000 resamples were used for these analyses.

### Bioinformatic and statistical analyses

TMB assessment was conducted during the published clinical trial (Rizvi et al. 2018), and the exploration of the relationships between ARID1 subunits and immunocytes derived from the TIME was carried out with an online tool (Ru et al. 2019). All statistical analyses were conducted by GraphPad Prism 8.0 software (GraphPad, La Jolla, CA), and Student’s t tests were used to determine statistical significance. *P* values were determined by two-tailed tests, and *P* < 0.05 was considered statistically significant.

## Results

### The prevalence of mutant *ARID1* and its role in the prognosis of NSCLC

**Fig. 1:**
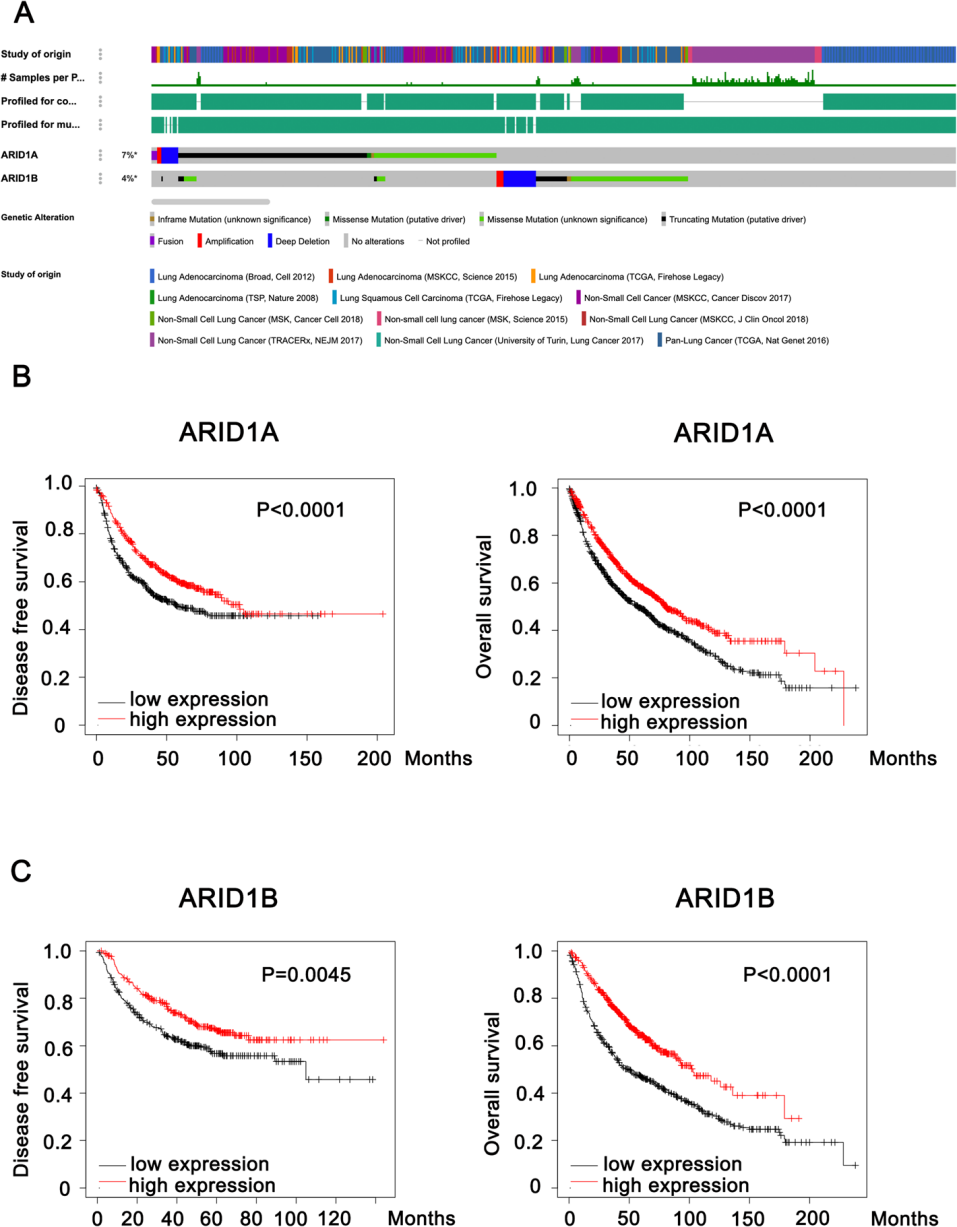
ARID1 subunits are tightly associated with the prognosis of NSCLC. **a**. The prevalence of ARID1 subunits mutations in NSCLC patients according to the cBioPortal for Cancer Genomics; **b**. The relationship between ARID1A expression and the prognosis of NSCLC patients; **c**. The relationship between ARID1B expression and the prognosis of NSCLC patients

According to the datasets acquired from the cBioPortal for Cancer Genomics, *ARID1* mutation is common among NSCLC patients. As shown in Fig. 1a, the mutation frequencies of the subunits of *ARID1* including *ARID1A* and *ARID1B* were 7 and 4% in NSCLC patients, respectively. As far as we are concerned, gene mutations or hypermethylation lead to low ARID1 protein expression (Guan et al. 2011; Zhang et al. 2013), therefore, we further investigated the relationship between the survival of NSCLC patients and the expression of the ARID1 protein. Figure 1b and c describe the relationships between the disease-free survival (DFS) or OS of NSCLC patients and the expression of ARID1A or ARID1B. As shown in the figure, ARID1A and ARID1B both are convincing biomarkers for NSCLC prognosis with compelling efficiency, and ARID1A or ARID1B deficiency was significantly related to the poor prognosis of NSCLC (ARID1A [DFS: *P* < 0.0001; OS: *P* < 0.0001]; ARID1B [DFS: *P* = 0.0045; OS: *P* < 0.0001]).

### *ARID1A* or *ARID1B* mutation correlates with an improved outcome for ICIs treatment

**Fig. 2:**
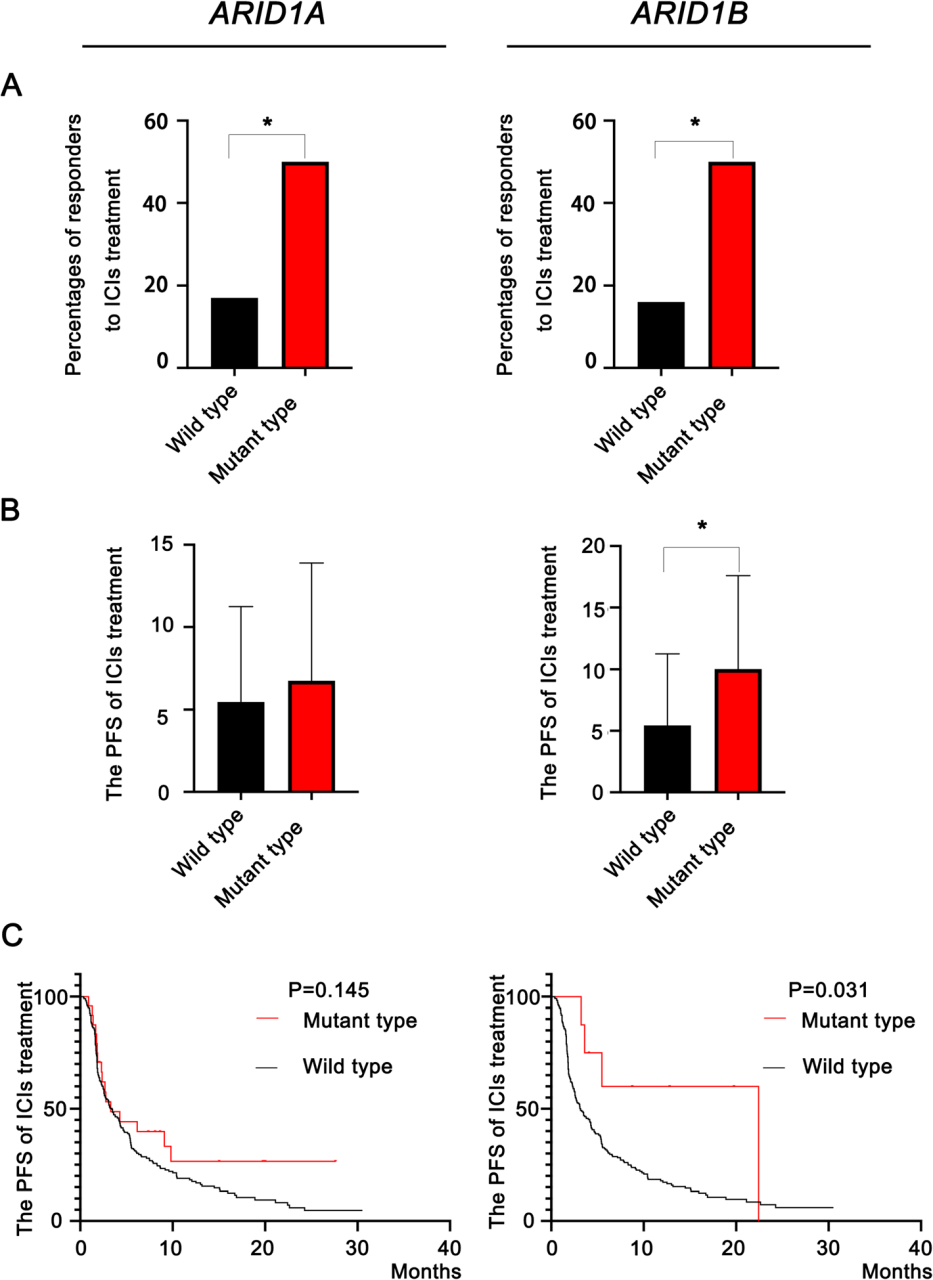
Mutations in ARID1 subunits predict an improved prognosis for cancer immunotherapy. **a** The different percentages of patients who responded (complete response+partial response+stable disease) to immune checkpoint inhibitors (ICIs) grouped by the genomic signatures of ARID1 subunits; **b**. The different progression-free survival (PFS) times grouped by the genomic signatures of ARID1 subunits; **c**. The survival curves for ICIs-treated patients based on the genomic signatures of ARID1 subunits. (*: *P*<0.05.)

The relationship between *ARID1A* or *ARID1B* mutation and the outcome of ICIs treatment was then studied. Through systematic analyses of the datasets from the cBioPortal for Cancer Genomics, we found that both *ARID1A* and *ARID1B* mutations were associated with an improved outcome for ICIs treatment in advanced NSCLC patients. As shown in Fig. 2a, more responders (CR + PR + SD) were confirmed in the mutant-type (MT) group than in the wild-type (WT) group for patients harboring *ARID1A* (50% versus 19%, *P* = 0.045) or *ARID1B* (50% versus 16%, *P* = 0.034) mutations. Figure 2b displays the median PFS (mPFS) with ICIs treatment for the two groups. Patients harboring *ARID1A* (6.8 months versus 5.5 months, *P* = 0.313) or *ARID1B* (10.0 months versus 5.4 months, *P* = 0.032) mutations benefited more from treatment and achieved a longer PFS time than those in the WT group. Survival analyses for ICIs treatment were then performed as shown in Fig. 2c. Compared with those in the WT group, patients harboring mutant *ARID1B* achieved significant survival benefits with treatment (*P* = 0.031). However, although a trend toward a difference existed for the survival curve of patients harboring mutant *ARID1A*, no statistical significance was found (*P* = 0.145).

### Establishment of a prognostic nomogram for the prognosis of ICIs treatment in NSCLC

**Fig. 3:**
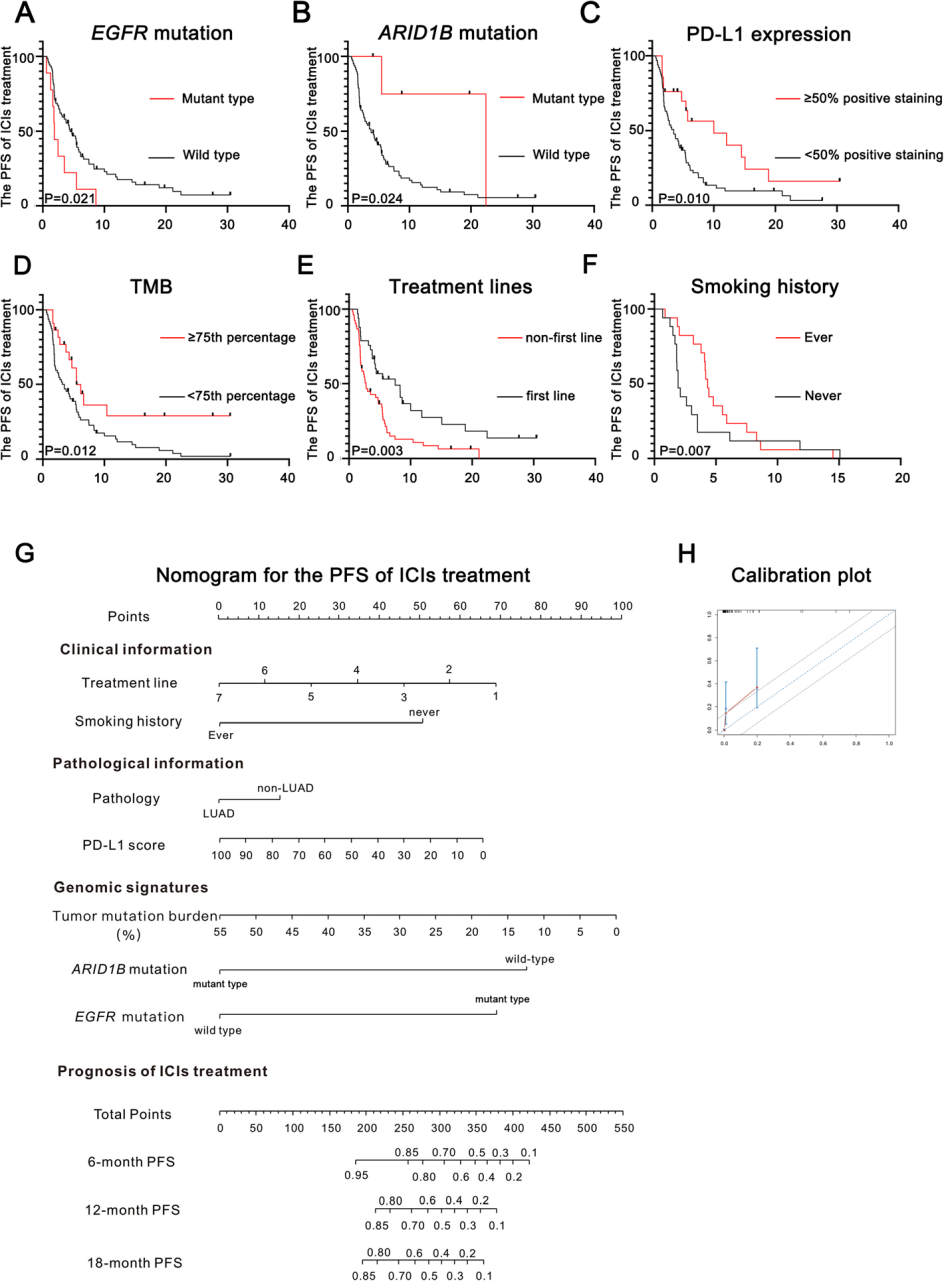
Novel nomogram to predict the prognosis of immune checkpoint inhibitor (ICI) treatment. **a** The survival curves for ICIs-treated patients based on the *EGFR* mutational status; **b**. The survival curves for ICIs-treated patients based on the *ARID1B* mutational status; **c**. The survival curves for ICIs-treated patients based on PD-L1 expression; **d**. The survival curves for ICIs-treated patients based on the tumor mutational burden (TMB); **e**. The survival curves for ICIs-treated patients based on treatment lines; **f**. The survival curves for ICIs-treated patients based on smoking history; **g**. The novel nomogram based on patient information to predict the prognosis of ICI treatment; **h**. The calibration plot for the nomogram

Eighty-six patients of the selected NSCLC cohort with integrated information on clinical features, targeted sequencing and PD-L1 expression evaluated by immunohistochemistry (IHC) were involved in the construction of the novel nomogram. First, univariate analyses were performed to identify variables to include in nomogram construction. As shown in Fig. 3a to f, multiple variables were confirmed to be significantly associated with the prognosis of ICI treatment, including *EGFR* mutation (*P* = 0.021), *ARID1B* mutation (*P* = 0.024), PD-L1 expression (*P* = 0.010), TMB (*P* = 0.012), treatment lines (*P* = 0.003) and smoking history (*P* = 0.007). Through the univariate analyses, we found that patients with mutant *ARID1B*, elevated PD-L1 expression (≥50% percentage positive staining), a high TMB value (≥75th percentage) or a history of smoking could benefit from ICIs treatment, while patients harboring mutant *EGFR* might not derive survival benefits from ICIs treatment. In addition, first-line administration of ICIs in advanced NSCLC patients might be a better choice than later administration. The nomogram based on these variables was then established as shown in Fig. 3g. In total, 3 types of patient information, including the clinical information, pathological information and genomic signatures, were included in the nomogram. Through this novel nomogram, physicians could easily obtain a score based on the Cox regression model for each variable listed in the graph, and then the total number would be assessed as the sum of all variable scores. Therefore, the survival risks of ICIs treatment for advanced NSCLC patients could be quantified before treatment. The C-index for this prognostic model was 0.71, which suggests that the model has a relatively robust ability to predict the PFS of advanced NSCLC patients treated with ICIs. The calibration plots shown in Fig. 3h indicated that the probabilities of our prognostic model agreed with the accuracy probabilities on acceptable scales (the dashed lines in the calibration plots correspond to a 10% margin of error).

### The mutation status of *ARID1A* or *ARID1B* is associated with the TMB level, PD-L1 expression and the TIME modulation of NSCLC

**Fig. 4:**
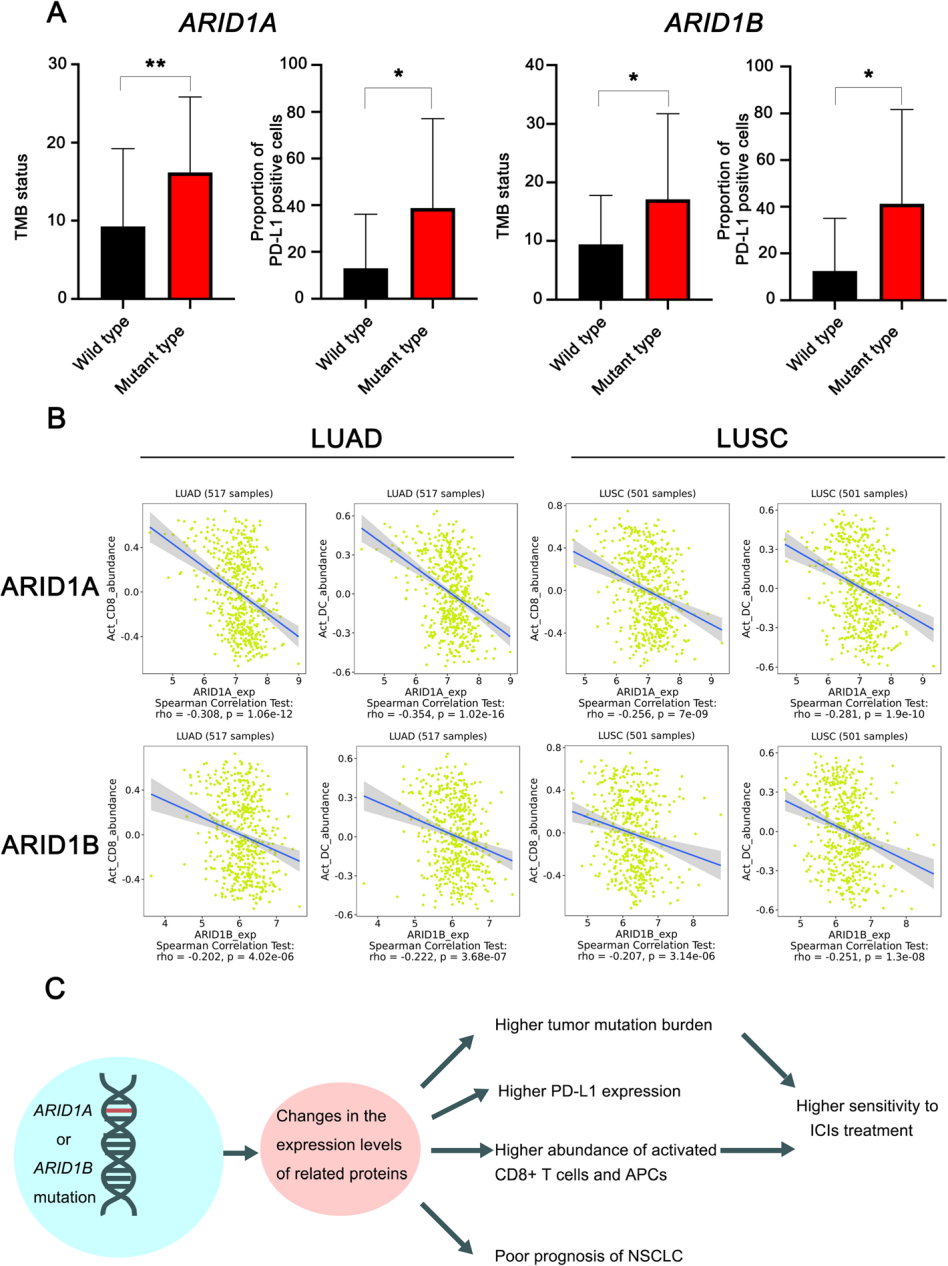
*ARID1A* or *ARID1B* mutations are tightly associated with sensitivity to immune checkpoint inhibitors (ICIs). **a**. The comparison of tumor mutational burden (TMB) values and PD-L1 expression grouped by the genomic signatures of ARID1 subunits; **b**. The correlations between ARID1A or ARID1B expression and the abundances of activated CD8+ T cells and activated dendritic cells (DC) in lung adenocarcinoma (LUAD) and lung squamous cell carcinoma (LUSC); **c**. The underlying relationships between prognosis and *ARID1A* or *ARID1B* mutation deduced from this research. (**: *P*<0.01; *: *P*<0.05)

Based on the research above, we reasonably deduced that *ARID1A* or *ARID1B* mutation serves as a novel biomarker for ICIs treatment and could have a connection with factors that are proven to be associated with sensitivity to cancer immunotherapy. As shown in Fig. 4a, *ARID1A* or *ARID1B* mutations were associated with a higher TMB value (*ARID1A*: 16.2 versus 9.3, *P* = 0.001; *ARID1B*: 17.1 versus 9.4, *P* = 0.020) and a higher proportion of PD-L1-positive cells (*ARID1A*: 38.9% versus 12.9%, *P* = 0.040; *ARID1B*: 41.3% versus 12.4%, P = 0.020) in advanced NSCLC patients. Figure 4b reveals the relationships between the TIME with the expression of ARID1A or ARID1B. As shown in the figure, ARID1A or ARID1B expression was related to immunosuppression in 517 lung adenocarcinoma (LUAD) samples and 501 lung squamous cell carcinoma (LUSC) samples, which is mainly characterized by significant reductions in the abundances of activated CD8+ T cells and activated dendritic cells (DCs). This result suggested that ARID1A or ARID1B deficiency might modulate the TIME via the activation of the antigen presentation process and cellular immunity and thus contribute to the change in the sensitivity to ICIs treatment.

## Discussion

As is well known, the PD-L1 expression or TMB level did not disclose satisfied efficiency in selecting patients who might benefit from immunotherapy while plasma NGS circulating-free DNA (cfDNA) analysis in NSCLC might provides new biomarkers for cancer immunotherapy (Rossi et al. 2020). According to the previous studies, the co-mutations of *TP53* and *STK11* serve as an important role in modulating the TIME of NSCLC and related to the sensitivity to ICIs treatment (Skoulidis and Heymach 2019). Especially for *STK11* and *KEAP1* mutations, which were found more prevalent altered in *KRAS*-mutated LUAD, demonstrate their roles in inducing the primary resistance to ICIs treatment (Skoulidis and Heymach 2019; Skoulidis et al. 2018; Papillon-Cavanagh et al. 2020). On the contrary, mutations of *ARID1A*, which belongs to the ARID1 family member, might be associated with a different outcome among patients received ICIs treatment and participate to the improved ICIs outcome in NSCLC via associating *KRAS* mutations as reported by Gandara D, et al. Besides, Goswami S, et al. confirmed that *ARID1A* mutations plus CXCL13 expression as combinatorial biomarkers to predict the sensitive phenotype to ICIs in metastatic urothelial carcinoma (Goswami et al. 2020). In addition, the NGS examination based on MYSTIC trial (Rizvi et al. 2020) revealed that *ARID1A* mutations are associated with improved outcomes with the PD-1 inhibitor durvalumab plus the CTLA4 inhibitor tremelimumab reported by Rizvi NA, et al. Based on these studies for new biomarkers of cancer immunotherapy especially for *ARID1A* mutations, we conducted our research in order to further investigate the significance of *ARID1* mutations, including *ARID1A* and *ARID1B*, in predicting the prognosis of ICIs treatment for advanced NSCLC.

To the best of our knowledge, our research is the first to illustrate the roles of the ARID1 subunits in the prognosis of cancer immunotherapy and to establish a prognostic model for immunotherapy in advanced NSCLC patients. Through this research, the roles of ARID1A and ARID1B in the prognosis of NSCLC were clearly clarified, and both ARID1A deficiency and ARID1B deficiency were related to the poor prognosis of NSCLC patients. However, *ARID1A* or *ARID1B* mutations and the resultant functional deficiencies were tightly associated with the sensitive phenotype for cancer immunotherapy. Patients harboring *ARID1A* or *ARID1B* mutations were more likely to have a good response to treatment with ICIs, and their PFS time might be prolonged through ICIs treatment. These correlations were also verified by the prognostic nomogram based on the Cox regression model established in this research. Here, we provided patients and physicians with this novel nomogram to estimate the survival risks of ICI treatment and determine an appropriate regimen for treatment and follow-up before treatment is initiated.

The results from the latest studies suggest that *ARID1A* mutation and the resultant functional deficiency are associated with the elevation in PD-L1 expression and the TMB value in ovarian cancer (Shen et al. 2018) and gastric cancer (Li et al. 2019), which indicates the underlying relationship between ARID1A and potential therapeutic anticancer immunity. Whether this correlation can be verified in NSCLC and the potential role of ARID1B in cancer immunotherapy remain to be further studied. The bioinformatic analyses in this research indicated that *ARID1A* or *ARID1B* mutations were associated with instability in the cancer cell genome and cancer mutability, as indicated by the elevated TMB, which is a proven biomarker for cancer immunotherapy. Patients harboring *ARID1A* or *ARID1B* mutations are likely to exhibit enhanced expression of PD-L1. In addition, through the activation of the antigen presentation process and anticancer cellular immunity, ARID1A and ARID1B deficiencies could modulate the TIME of NSCLC and initiate a therapeutic immune reaction to tumors. In this research, we confirmed the important role of ARID1A deficiency in elevating cancerous mutability and changing tumors to exhibit an aggressive phenotype in NSCLC, which was proposed by published works (Shen et al. 2018; Jiang et al. 2020; Okamura et al. 2020; Rossi et al. 2020) on other cancer types. Additionally, our research is the first to investigate the role of ARID1B, which is another ARID1 subunit similar to ARID1A, in changing the phenotype of cancer. Given the results reported in this research, we proposed that ARID1A and ARID1B might be equally important in cancer immunotherapy and the prognosis of NSCLC.

As shown in Fig. 4c, we generated new results in this research and reasonably deduced that mutation of *ARID1A* or *ARID1B* leads to changes in the expression levels of related proteins, which results in the poor prognosis of NSCLC on the one hand but enhances the sensitivity to ICI treatment in advanced NSCLC through elevating the TMB, enhancing PD-L1 expression and modulating the anticancer immune reaction on the other hand. Admittedly, our research might have several limitations. Further investigations into the role of ARID1B in cancer immunotherapy are needed, given the small sample size in this research. Although the results suggest that ARID1A and ARID1B may participate in the modulation of the TIME and are associated with the elevations in tumor mutability and PD-L1 expression, no molecular mechanism was explored in this research. Further studies are needed to explore the mechanism underlying these correlations.

## Conclusion

ARID1A and ARID1B could serve as novel biomarkers for the prognosis and sensitivity to ICIs treatment of advanced NSCLC. *ARID1A* or *ARID1B* mutations and the resultant functional deficiencies are tightly associated with cancer mutability, PD-L1 expression and TIME modulation and are associated with a good prognosis for ICIs treatment. The relationship between the prognosis of ICIs treatment and *ARID1B* mutation could be verified by the novel nomogram for ICIs treatment.

## Data Availability

All data generated during this study are included in this published article. The datasets generated in the current study are available in the cBioportal for Cancer Genomics [Cerami et al. 2012; Gao et al. 2013] (http://www.cbioportal.org/).

## Abbreviations

SWI/SNF: Switch/sucrose nonfermenting
ARID1A: AT-rich interactive domain 1A
ARID1B: AT-rich interactive domain 1B
BAF: BRM-associated factor
NSCLC: Non-small cell lung cancer
EGFR: Epidermal growth factor receptor
ALK: Anaplastic lymphoma kinase
IHC: Immunohistochemistry
DFS: Disease-free survival
ICIs: Immune checkpoint inhibitors
PD-L1: Programmed death-ligand 1
TMB: Tumor mutational burden
